# What is required for community-based participatory research to be effective? Studying asylum-seekers’ access to healthcare

**DOI:** 10.1186/s13584-018-0202-7

**Published:** 2018-01-05

**Authors:** Ifat Vainer, Tamy Shohat

**Affiliations:** 1 Israel Center for Disease Control, Israel Ministry of Health, Gertner Institute, Sheba Medical Center, Ramat- Gan, Israel; 2 Department of Epidemiology and Preventive Health, Sackler Faculty of Medicine, Tel Aviv University, Tel Aviv, Israel

**Keywords:** Community-based participatory research approach, Asylum seekers, Healthcare

## Abstract

This commentary on the paper by Gottlieb et al. [IJPHR November 2017] expands the discussion of the community-based participatory research approach, which is based on equal partnership between researchers and community members. We emphasize the essential principles that were originally introduced in order for this approach to be effective.

## Commentary

With the increasing number of displaced persons worldwide, health care systems are faced with the challenge of providing health needs to these heterogeneous populations. Asylum seekers are at increased risk of having several health problems, and of having problems of high severity [1]. However, in many countries, access to health care for these people is restricted to emergency care only [2].

The definition of displaced persons varies between countries. While refugees and asylum seekers predominate, access issues in healthcare are relevant to a much larger populace of authorized and unauthorized labor migrants. In 2016, about 42,000 asylum seekers and over 90,000 unauthorized workers were estimated to be living in Israel [3]. In the current situation these populations are not included under the National Health Insurance Law. Certain public health services such as those for pregnant women and children and the diagnosis and treatment of sexually transmitted diseases and tuberculosis are free of charge. Emergency care is available to all, yet it is not free, so hospitals may later demand payment. However, primary care, which is the service most in demand, is offered by two volunteer based walk-in clinics in Tel- Aviv only. The services provided in both clinics are limited. Private health insurance is theoretically available but is expensive [1].

In an article recently published in the IJHPR, Gottlieb et al. [4] reported the results of the first attempt to apply a community-based participatory research (CBPR) approach to migrant health research in Israel [4]. That pilot study was conducted among Eritrean asylum seekers in four Israeli cities. The goals were to generate data that may help health policymakers better understand Eritrean health needs, to test the feasibility and added value of CBPR and to support the collective efficacy and social inclusion of asylum-seeking communities. The study team consisted of two local activists, a public health nurse and four Eritrean community partners. A 22-item survey was designed and administered by eight community members who were recruited for that purpose. Each surveyor was asked to approach 100 Eritrean asylum seekers above the age of 17. Six follow-up interviews and two focus group discussions with Eritrean community members contributed to the interpretation of the survey findings. The majority of the Eritrean asylum seekers (95%) expressed interest in joining a health insurance scheme. More than half were willing to pay a monthly sum of 100–300 NIS for health insurance and an additional 40% were willing to pay up to 100 NIS. The authors concluded that the study provided initial evidence of asylum seekers’ willingness to pay for a public health insurance plan, supports the potential of CBPR in enhancing research of marginalized populations, and counters their social exclusion through capacity building.

The authors should be congratulated for this first attempt to apply a CBPR approach to a migrant population in Israel. They succeeded in accessing information from asylum seekers in a number of cities in Israel and in gaining their trust. CBPR has become a popular tool to study migrant and marginalized populations. It engages immigrants in all aspects of the research: development of the research questions, data collection and interpretation, and dissemination of results. An increasing number of research studies have utilized this methodology for exploring complex health issues for immigrants [5]. CBPR emphasizes the strengths and resources of every partner by valuing co-research, empowerment and capacity building, combining knowledge, and bi-directional leadership and decision-making [6]. This approach contrasts sharply with traditional research, in which the academician is the expert who conducts research, with little or no input from the participants or community under study [7, 8] .

Barbara Israel, one of the most experienced researchers in conducting CBPR, has suggested nine principles that characterize this type of research. Israel and colleagues have emphasized the importance of community participation, and the need for cooperation and co-learning among professional and community researchers; the aim should be the establishment of effective and sustainable partnerships between professional and community researchers [9, 10]. CBPR entails partnership in all phases of the research. Moreover, integral to CBPR is an intensive preparatory stage within the community where the study is carried out. The researchers involved in the project are expected to have special training, which fosters self-reflectiveness, humility and the willingness to admit to making mistakes, good communication skills, interpersonal and facilitation skills, and connection and sensitivity to the needs of the community [11].

Gottleib et al.’s study seems to lack some of the desired principles of CBPR. For one, the academic partner is not completely clear [12]. This may be because the study was not originally designed to use CBPR methods. When the academic nurse with expertise in CBPR joined the team, the study was already underway. The survey questionnaire was developed by the team members; they indeed included four Eritrean community partners; however, it is not clear how these individuals were chosen and whom they represented. Involvement of members of the community in the planning process and the development of the survey seemed limited. The fact that community members collected the data is not enough to satisfy requirements for CBPR. The surveyors, all males, were trained during 2 3-h sessions. Two main challenges of CBPR are to recruit a sufficient number of respondents for the research from the investigated community and to recruit individuals who represent the community [9]. In the present study, the response rate was 40% and the respondents were a convenience sample. Dissemination of the results to all partners and involving them in this process [13], as well as a commitment to sustainability are another two principles of CBPR that were not completely met in the present study.

Despite the above limitations, Gottleib et al.’s study is of great value. It is the first attempt to use a CBPR initiative for studying asylum- seekers in Israel. The authors succeeded in directly involving the community partners in some aspects of the project. They also succeeded in maintaining some follow- up discussions.

A recent review of immigrants in CBPR examined the extent to which the study populations were involved in the research. The results of this review demonstrated that in just over half of 161 articles (52%) immigrants were directly involved in the CBPR project [14]. CBPR demonstrated greater potential impact on populations when immigrant involvement was more comprehensive.

In conclusion, CBPR affords the opportunity to establish a relationship between academic research and communities, provided it is carried out according to the established principles [9]. Encouragement should be given to further studies that partner researchers and various groups of immigrants residing in Israel, in light of the current problematic situation of the latter regarding proper access to health services.

## Abbreviation

CBPR: Community-Based Participatory Research
